# Influence of Protic Ionic Liquid-Based Flame Retardant on the Flammability and Water Sorption of Alkalized Hemp Fiber-Reinforced PLA Composites

**DOI:** 10.3390/polym15183661

**Published:** 2023-09-05

**Authors:** Percy Festus Alao, Raimond Press, Jussi Ruponen, Valdek Mikli, Jaan Kers

**Affiliations:** 1 Department of Material and Environmental Technology, Tallinn University of Technology, Ehitajate tee 5, 19086 Tallinn, Estonia; rapres@taltech.ee (R.P.); jaan.kers@taltech.ee (J.K.); 2 Department of Bioproducts and Biosystems, Aalto University, Vuorimiehentie 1, FI-00076 Espoo, Finland; jussi.ruponen@aalto.fi; 3 Department of Polymer Materials, Tallinn University of Technology, 19086 Tallinn, Estonia; valdek.mikli@taltech.ee

**Keywords:** hemp fibers, fire-retardant fiber-reinforced composites, fire reaction, hygroscopic, fire classification, transportation materials

## Abstract

This article investigates the effects of combining a novel protic ionic liquid-based fire retardant (FR) with alkalized hemp fiber. A pivotal importance of this study refers to the hydrophilic properties and limits regarding poor thermal resistance of green composites where standard guidelines for fire risks are crucial. Although it is well-studied that alkalization is essential for green composite’s moisture and mechanical durability, research on the flammability of such a combined treatment for natural fiber-reinforced biopolymer composites is lacking. The alkaline treatment used in the current study follows a process already studied as optimal, particularly for the selected hemp fiber. The fire performance was examined using a bench scale approach based on self and piloted ignition from cone calorimeter tests. The result from the Fourier-transform infrared analysis of the hemp fiber confirms phosphorylation following the fire-retardant treatment, which was visible from the morphological examination with scanning electron microscope. The presence of FR in the composites led to impactful moisture sorption. However, the FR composites demonstrated an enhanced response to fire, indicating potential use as a Class B standard for building construction, and hazard level 3 (HL3) classification as an interior material in vehicles, provided the problem of high emission of smoke is mitigated.

## 1. Introduction

In temperate climates, such as Europe, hemp is regarded as one of the best options for obtaining natural fibers, since it is subject to European Union agricultural subsidies [1] and has a very reasonable price, particularly when compared to synthetic fibers [2]. Although flax is still the most extensively used natural fiber because of its superior mechanical qualities and heat resistance, expense is a significant consideration when selecting fibers for some purposes. Hence, hemp fibers are more competitive when price is a main consideration [3]. Furthermore, compared to flax and the majority of other natural fiber sources, hemp plants have less of an impact on the environment during cultivation and have fewer nutritional and development requirements [4].

Natural fibers have potential applications in the automotive and construction industries, as well as for packaging and retail. However, the widespread use of natural fibers is hampered by several aspects, including high flammability and hydrophilic properties, which cause problems, such as moisture affinity and unsatisfactory interfacial adhesion between matrix and fiber, limiting mechanical performance and the ability to meet specific application standards [5]. Regardless, while mechanical and physical properties are vital, the thermal stability of bio/green composites remains of critical interest for a wide range of applications, as does the availability of the natural material in the local region and the optimal price–performance ratio. The hydrophilic properties of natural fibers, like hemp, have been studied through chemical treatment to manage the amorphous composition, which also contributes to the mechanical performance of composites [6,7,8,9,10,11]. However, the limitation still arises with the fiber chemical treatment, since most of the processes do not consider the chemical composition of the natural fiber [9], which is dependent on growth and retting circumstances [2]. In terms of fire protection, natural fibers contribute to the heat conductivity of polymer composites, making them highly combustible. As the usage of such materials grows in popularity and their poor thermal qualities represent a risk, improving their inflammability is crucial [12].

Although fire retardants (FR) have been widely used to improve thermal stability, there has been little research on natural fiber/hemp fibers, particularly when combining chemical surface modification with FR. Furthermore, there are no known fire performance classifications (Euro class) to offer data on such combo treatment. Furthermore, studies have shown that chemical treatment of natural fibers can cause increased flammability, because the reduction in amorphous content in the fiber results in more flammability due to the formation of more levoglucosan [5]. Besides, lignin and ash contents are essential to enable increased char formation during burning. Additionally, a combination of better fiber orientation and higher crystallinity affects the composite pyrolysis differently. Natural fibers disintegrate under sufficient heat to produce combustible and non-flammable gases, tar, and char. However, because of the high variability of natural fibers, the reaction to fire of the composite is reliant on the type of fiber used as reinforcement [13]. For example, a previous study on the hemp fiber used in the current research found no significant difference in the mass loss, ignition time, temperature transfer through depth, basic protection time, or the start of char time for the composites with either untreated or alkali modified fibers [14], which is primarily due to the hemp fiber’s inherently low lignin content (1.4%) [15]. Despite this, considerable improvements in the mechanical characteristics [15,16], water absorption, and hygroscopic properties [14] of the composites were documented following alkali treatment. The biggest issue due to fire-retardant treatments of natural fibers is the loss of mechanical qualities [16]. As a result, the challenge is to treat the fibers in a way that delivers both sufficient reaction to fire and mechanical and water sorption durability. The composite structure should not be compromised, and the adhesion between the fiber and the matrix should not deteriorate to achieve good mechanical characteristics of biocomposites.

The current study focuses on identifying flame propagation capability, fire classification, particularly applicable to the transportation industry, and the impact of fire-retardant treatment on moisture absorption of alkalized hemp fiber-reinforced polylactic acid (PLA) composites. Combining natural fiber and polymers from renewable sources is essential for achieving significant environmental sustainability. PLA provides such a possibility; however, processing challenges, poor thermal stability, and poor flame retardancy are limitations for several applications. Regardless, PLA is still the most suitable choice of polymer for a fully biobased composite due to its extensive commercial availability compared to currently available renewable alternatives and its use of 55% less manufacturing energy than polymers made from petroleum [17]. Palonot Oy fire retardants (P_2_ (Palonot 2) and P_4_ (Palonot 4)) were examined for this purpose. P_2_ and P_4_ are novel protic ionic liquid-based fire retardants (FR). Ionic liquids (ILs) are solutions that contain a significant number of ions. Protic ILs are a characteristic category of ILs obtained as products of an acid-base neutralization reaction. The ignition parameters and mechanical performance of composites with chemically modified hemp fibers and a P_2_ FR have previously been reported [16], but the impact of these combined treatments on moisture resistance and the fire class of the green composite has yet to be determined. The Palonot FR has not been extensively studied for hemp fiber treatments, making it challenging to perform the full-scale fire resistance test based on a single burning item (SBI) to issue a certificate of fire performance in Europe for such composites as construction or building elements. According to Naughton et al. [18], SBI tests are a barrier to new product research and development since they only provide pass/fail information, and further scientific analysis of failure processes is limited because the samples are often destroyed during the test. Therefore, a cone calorimeter test approach (ISO 5660-1:2015 [19]) is a more precise tool for product development and quality control. Furthermore, the test is an ideal performance-based bench-scale fire testing method providing results for an exact description of the materials’ properties, such as heat release rate (HRR), peak heat release rate (pHRR), ignition times, and effective heat of combustion (EFC), and so on [20]. Moreover, the transportation sector, especially with regards to railway vehicles, uses guidelines according to EN 45545-2 [21] defined by the MAHRE (maximum average heat rate of emission) obtained from cone calorimeter tests (ISO 5660-1).

## 2. Materials and Methods

### 2.1. Materials

Frost-retted hemp fiber (*Cannabis sativa*, dioecious Hungarian variety Tisza) with a density of 1.26 gcm^−3^, staple polylactic acid (PLA) [NatureWorks LLC] with a density of 1.24 gcm^−3^ as matrix, NaOH (CAS: 1310-73-2: “STANCHEM” Sp. Z o.o, Boduszyn, Poland) for the fiber surface treatment, and two variants of fire retardants (P_2_ and P_4_) from Palonot Oy (Tekniikantie 2, Espoo, Finland) were used. The property of the FR was previously described in a recent publication [16]. The Palonot FR is essentially composed of an aqueous solution of bisphosphonate acid, an alkanol amine, and, optionally, an alkaline ingredient that provides extra defense against mold, rot, blue stain fungus, damage by insects, and dimensional changes, and is non-hazardous to human health. The P_2_ and P_4_ categories of Palonot F1 differ by slight alterations in the concentrations of the intrinsic components.

### 2.2. Methods

#### 2.2.1. Fiber Pretreatment (Alkali) and Fire Retardant

Fiber pretreatment and fire-retardant treatment follow the process already described by Alao et al. [16]. The carded hemp fibers were dried in the oven at 80 °C for 24 h and then optimally alkalized. Alkalization was performed using a 5 wt.% NaOH solution for 60 min because this pretreatment concentration and time is observed to be the most effective for the hemp fibers [16]. For FR treatment, an average of 22 mL of the Palonot FR was diluted to 50 mL with distilled water before spraying on the hemp mats.

#### 2.2.2. Composite Fabrication

Table 1 shows the batch of composites fabricated. Both NaP_2_ and NaP_4_ were produced and compared to Na (composites of NaOH treated hemp fiber, without FR). The variants with unmodified hemp fiber (UTP_2_ and UTP_4_) were not the focus of this research because the properties of UTP (untreated hemp fiber composite with Palonot F1) have already been examined with the NaP (alkalinized variant), particularly regarding self-ignition, which proved that combination treatment offers improved FR composite fire performance. However, for certain characterizations, samples with non-alkalized fibers were also assessed. All batches of the composites were produced using 50 wt.% unidirectional (aligned in a single direction) hemp fibers and PLA matrix. Additionally, the composite variants with FR were fabricated following the methods described in a previous study [16]. The composites with FR had approximately 24% more density, indicative of FR retention. To better describe some aspects of the composite properties, the untreated variants of the composite with FR are also examined.

#### 2.2.3. Scanning Electron Microscopy (SEM)

SEM was conducted using a high-resolution Zeiss Ultra 55 (FELMIZFE, Graz, Austria) scanning electron microscope on cross sections prepared from the composites. The SEM was operated at a voltage of 20 kV, a scanning depth of up to 100 nm, and magnification of about 50,000.

#### 2.2.4. Fourier-Transform Infrared (FTIR) Spectroscopy

FTIR spectroscopy was performed with a Shimadzu IRAffinity-1S (Tokyo, Japan) infrared spectrometer obtained from Shimadzu Europa GmbH (Duisburg, Frankfurt, Germany) to assess the changes in the structural properties of the fiber following FR deposition. The samples were conditioned for 7 days prior to the analysis. A background scan of a clean Zn–Se diamond crystal was performed at a resolution of 4 cm^−1^ and range of 500–4000 cm^−1^. A total of 30 scans were recorded.

#### 2.2.5. Fire Behavior

**Figure 1:**
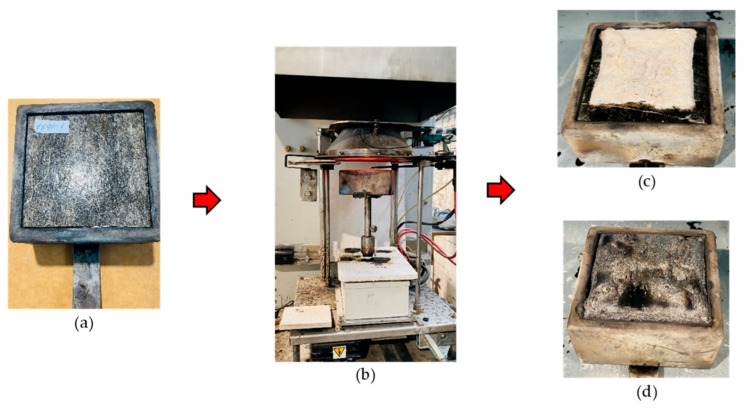
Cone calorimeter fire testing: (**a**) hemp composite in the sample holder; (**b**) piloted ignition testing with a spark ignition; composite after the test: (**c**) without FR; (**d**) with FR.

The fire behavior of the composite specimen was performed using a cone calorimeter (ISO 5660-1:2015), as shown in Figure 1. For the test, samples (100 mm × 100 mm) were subjected to an irradiance of 50 kWm^−2^ for 600 s using a cone heater at 25 mm from the exposed surface. Specimens were conditioned at a temperature of 23 °C and relative humidity of 50% for at least 7 days before the test. In certain cases, self-ignition was performed under 600 s, especially for specimens with low thermal stability (non-FR composites).

##### The Test According to ISO 5660 was Performed in Two Labs

*Self-ignition test*: Conducted in Tallinn University of Technology to access the surface temperature and temperature response through depth, $T_{\mathrm{depth}}$ (temperature on the unexposed face) of the specimen, measured by attaching 0.25 mm diameter type K thermocouples (Pentronic AB, Sweden) at the center point (50 mm). The $T_{\mathrm{depth}}$ is critical to obtain the protection time $T_{\mathrm{pt}}$ and start of char time $T_{\mathrm{ch}}$, which correspond to the temperatures of 270 °C and 300 °C (EN 1995-1-2:2004 [22]), respectively, for the timber placed under the composite. The experimental outcome is presented as a temperature–time curve measured at 3 s intervals. The result presents the composite’s ability to function as an effective fire barrier, especially to prevent flame spread to the timber.

*Piloted ignition*: Conducted in Forest and Wood Products Research and Development Institute (MeKA) fire testing laboratory (Jelgava, Latvia) to obtain necessary data, such as heat release rate (HRR)/total heat release (THR), limiting oxygen index (LOI), smoke production rate (SPR)/total smoke release (TSR), CO_2_ release, and mass loss. The nominal duct flow rate during the test was 24 ls^−1^. The exposed surface area was 88.4 cm^2^. Table 2 presents the physical properties of the investigated specimens.

Regarding the transportation sector, the maximum average heat rate of emission (MAHRE) from the ISO 5660-1 test procedure sufficiently classifies the composite performance according to requirements stipulated in EN 45545-2. Some of the categories within the testing framework, peculiar to the composite prospective application, are presented in Table 3. In view of the potential application of the composite in the transportation sector, applicable products mainly fall within requirement 1 (R1). The fire classification based on this standard uses hazard levels HL1, HL2 and HL3, which are requirement specific. HL3 represents the most demanding hazard level. It should be noted that some other requirements (including R19 and R21) may fall within the classification, especially because a lower heat flux (25 kWm^−2^) is required for the standardized test.

#### 2.2.6. Hygroscopic Properties

The hygroscopic behavior of the composites was examined according to EVS-EN ISO 12571:2021 [23] at a temperature of 23 ± 0.5 °C and relative humidities of 30%, 45%, 60%, 80%, and 95%. The composites were oven-dried to constant weight at 60 °C before the test in a conditioning chamber. The drying temperature of 60 °C was chosen to prevent any deformation to the polymer composite due to the low glass transition temperature of PLA (about 60 °C). The absorption and desorption equilibrium moisture contents ($\mathrm{EMC}\;\left(\%\right)$) were determined by weighing the mass of the specimens at equilibrium based on the following equation:

$$\mathrm{EMC}\;\left(\%\right)=\frac{m-m_{0}}{m_{0}}\times \;100$$

where $m_{0}$ is the mass of the oven-dried sample; m is the mass of the specimens at any given RH (relative humidity).

## 3. Results and Discussion

### 3.1. SEM

**Figure 2:**
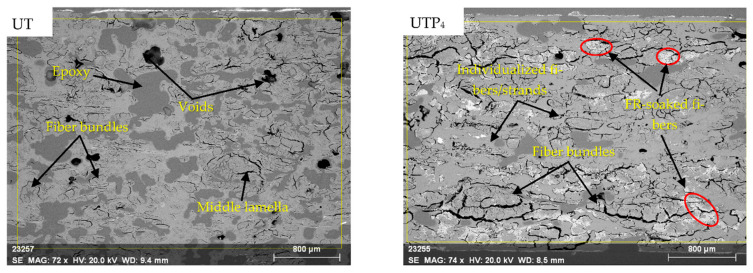

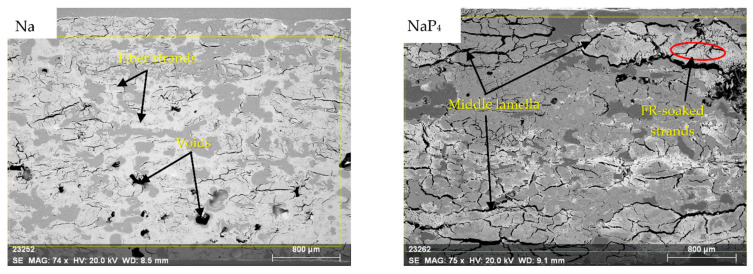
Cross-section SEM micrographs of composites of untreated hemp fiber (UT), alkalized hemp fiber (Na), untreated hemp fiber with fire retardant (UTP_4_), and alkalized hemp fibers with fire retardant (NaP_4_).

Figure 2 displays the cross-sectional SEM micrographs of the non-FR (UT and Na) and FR (UTP_4_ and NaP_4_, shown in this regard) composites. The epoxy glue used to embed all the samples before imaging appears as darker patterns (see Supplementary Data: Figures S1 and S2, Tables S1 and S2). The FR composites had different morphological characteristics from the other batch, including visible sections with more bundled fibers, pale fibers, and a center lamella. The differing manufacturing techniques and the retention of FR are the main reasons for these differences. Regardless, each batch of the composites contain voids, which are more evident in the non-FR composites. Although the fiber/PLA stacking technique is essential for the FR treatment, the presence of middle lamella in the images of FR composites indicates that PLA did not sufficiently penetrate the hemp fibers. The pale pigments on the hemp fibers indicate the presence of fire-retardant salt, confirmed by EDS analysis (Figures S3 and S4) as phosphorus, with normalized concentrations up to 12.5% (Table S3) and 15.7% (Table S4) for UTP_4_ and NaP_4_, respectively.

### 3.2. FTIR Analysis

**Figure 3:**
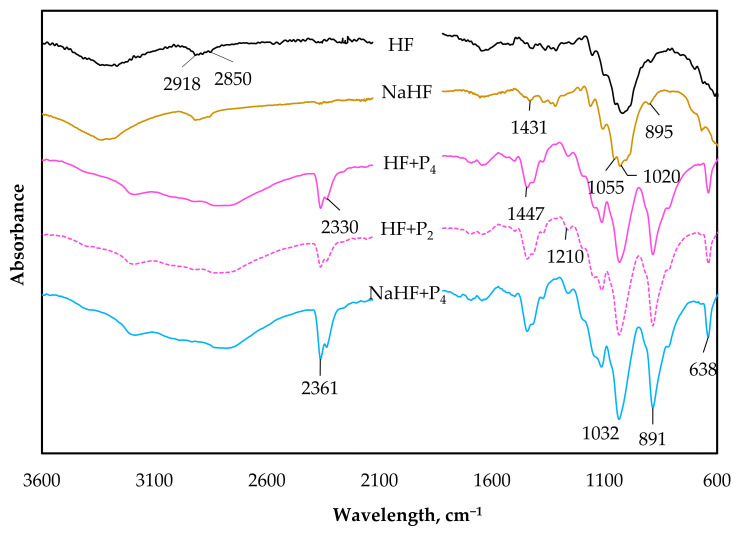
FTIR spectra of hemp fiber (HF), alkali modified hemp fiber (NaHF), and HF and NaHF with Palonot FR (HF + Palonot 2 (P_2_), HF + Palonot 4 (P_4_) and NaHF + P_4_).

**Figure A1:**
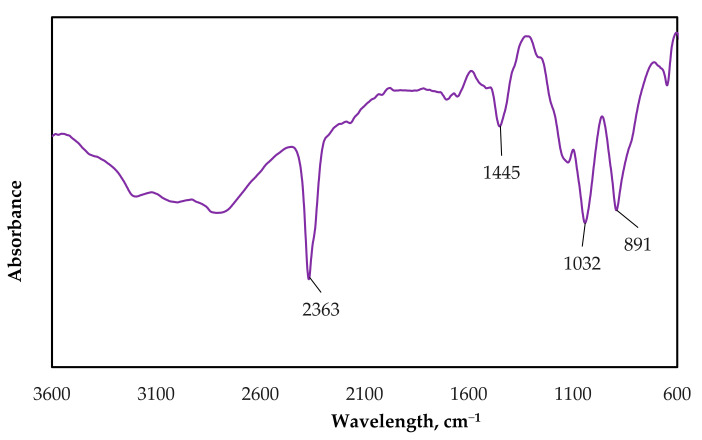
FTIR spectrum of alkalinized hemp fiber treated with Palonot 2 (NaHF + P_2_).

Figure 3 shows the characteristic peaks of the untreated hemp fiber (HF) and the impact of the NaOH treatment (NaHF), highlighted with peak subtraction/attenuation at 2850 cm^−1^, 2916 cm^−1^, 1235 cm^−1^, 1600–1650 cm^−1^, 1516 cm^−1^, and 1735 cm^−1^, accentuating the removal of/decrease in pectin, hemicelluloses, and lignin. The bands at around 1431 cm^−1^ and 895 cm^−1^ are affected by the fire-retardant treatment of both batches of hemp fibers (HF and NaHF), with new, intense peaks emerging from P−O−C bonds at 885–891 cm^−1^ and 1447 cm^−1^ [24]. Additional peaks at 1210 cm^−1^ and 1032 cm^−1^, respectively, are associated with P=O and P−O−C, while P−H bond-related peaks are observed at about 2361 cm^−1^ and 2330 cm^−1^. In addition, the bands corresponding to O−H stretching (3100–3500 cm^−1^) flattened. These findings show that the Palonot fire retardants caused the phosphorylation of the cellulose in the hemp fiber by modifying the CH glycosidic bond distortion relating to cellulose peak (around 895 cm^−1^), C−O stretching vibrations due to xylan and the glycosidic linkages of hemicellulose (1050 cm^−1^), and C−H bending of amorphous and crystalline cellulose (1430 cm^−1^). When comparing the two batches of FR (P_2_ and P_4_) under study, the variations in intrinsic composition and concentration did not present any apparent alterations in the peaks that had been identified. As a result, Appendix A, Figure A1, reports the FTIR spectrum of NaHF + P_2_, highlighting the similar peaks due to the phosphorylation of the alkalinized hemp fiber.

### 3.3. Reaction to Fire

#### 3.3.1. Self-Ignition

**Figure 4:**
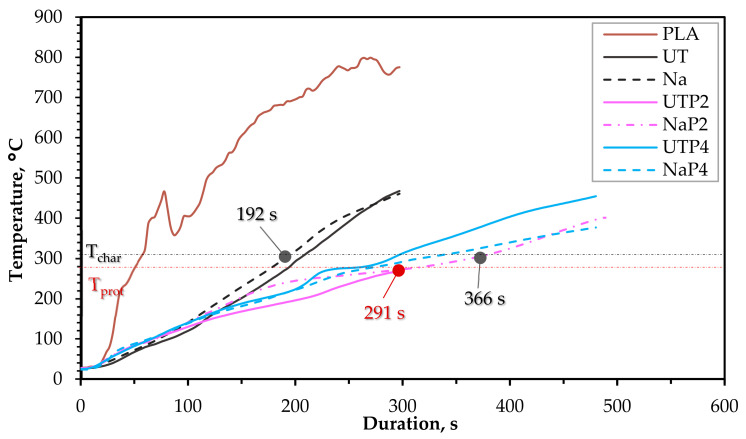
The temperature transfer through depth, measured on the interface between the composite and the timber block.

Figure 4 highlights the $T_{\mathrm{depth}}$ of the composites and PLA. A significant decrease in the $T_{\mathrm{depth}}$ is achieved by the composites containing the P_2_ and P_4_, clearly apparent from the increase in the $T_{\mathrm{pt}}$ and $T_{\mathrm{ch}}$ compared to the referenced non-FR specimens, which implies that the FR effectively improves the ability of the composites to serve as a fire barrier due to intumescence activation. The performance of P_2_ and P_4_ is alike, but P_2_ offers a slightly better result, e.g., the $T_{\mathrm{pt}}$ (291 s) and $T_{\mathrm{ch}}$ (366 s) of NaP_2_ were 34.5% and 15.1% better than NaP_4_, respectively. UTP_2_ also produced a similar outcome, with a 30% increase in $T_{\mathrm{pt}}$ compared to UTP_4_. Nonetheless, comparing the performance of P_2_ and P_4_ based on UT and Na did not show any substantial impact of the NaOH treatment on the functionality of the FR. The ignition time of Na and UT with the 50 wt.% hemp fiber at a cone heater distance of 25 mm is observed to be about 15 s. But the t_ig_ for FR composites (NaP_4_) in the current investigation are inconclusive due to a lack of ignition perceived in some repeats. This outcome is somewhat due to the nature of the test since pilotless ignition negates any medium to promote the combustion of combustible gases close to the surface of the specimens. Moreover, only the top layers of the composites are treated with FR. In some cases, the integrity of the composite is affected during the test, which causes the exposure of the core without FR coating, leading to an acceleration of the composite’s reaction to fire. However, the earliest t_ig_ was 141 s by a replica of NaP_4_, which represents a significant increase compared to Na.

#### 3.3.2. Piloted Ignition

The piloted ignition was only performed on the alkalized variants of the composite containing FR (NaP_2_ and NaP_4_). The composite of the alkali-treated hemp fiber without FR (Na) was also examined as a reference. This test was conducted to obtain essential reactions to fire parameters, such as CO_2_ emissions, pHRR, THR, MAHRE, and TSR.

**Figure 5:**
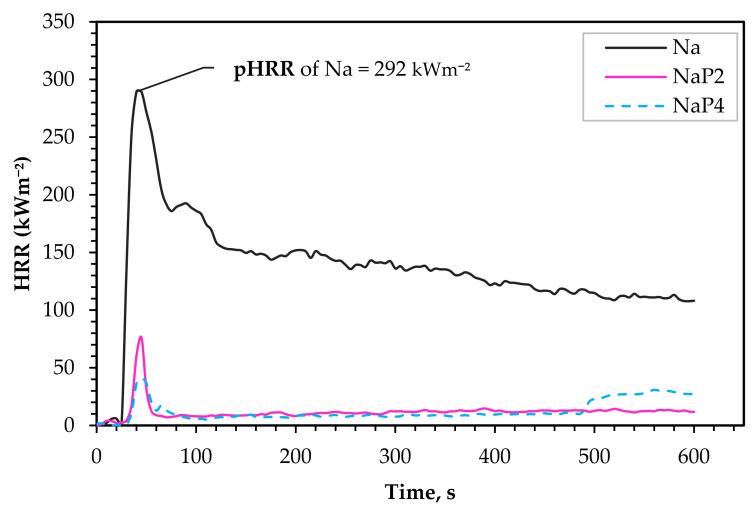
Heat release rate (HRR) profiles of Na, NaP_2_, and NaP_4_.

Figure 5 presents the average heat release rate curves for the composites. It is interesting to note that the t_ig_ of the Na composites was 8 s later than the t_ig_ during self-ignition. The ignition of Na causes a mean pHRR of 292 kWm^−2^ at about 43 ± 4 s. The HRR then reduces rapidly to 186 kWm^−2^, before a slight rise to 189 kWm^−2^, and then it stabilizes at 159–108 kWm^−2^. The double HRR peak is reportedly due to the subtle difference in combustion properties between the composite and the timber below the composite, which indicates flame spread to the timber, and the decomposition and production of combustible volatiles. This outcome is a sign of the non-FR composite’s inadequate fire barrier, which is consistent with past reports [5]. According to Janssen et al. [25], double peaks during such test are synonymous with wood-based products, and since the layer of composite serves as a fire barrier, the exposure of the underlying timber during test leads to an increase in the flammability parameters. Studies by Gallos et al. [26] and Hapuarachchi et al. [23], reported pHRR values for PLA at 342 kWm^−2^ and 485 kWm^−2^, respectively. The difference in pHRR is attributed to the heat flux used during the investigations: 35 kWm^−2^ [5] and 50 kWm^−2^ [27]. Regardless, comparing these pHRR values for PLA to Na in the current study signifies that hemp fiber reinforcement reduces the thermal decomposition of the polymer, which agrees with initial findings [16]. Additionally, the study [5] demonstrated that reinforcement with fibers and higher fiber content [27] improves the poor thermal performance of polymer matrices, like PLA.

**Figure 6:**
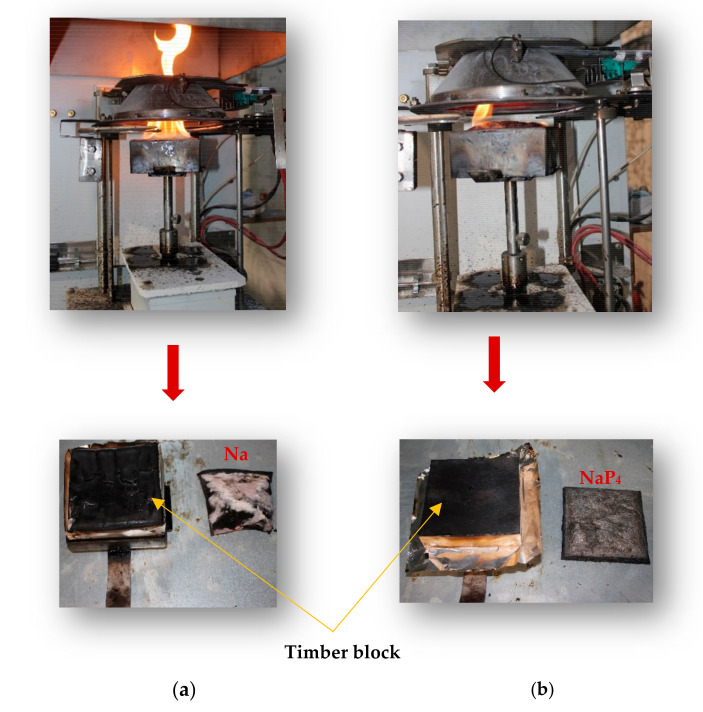
Piloted ignition testing: (**a**) ignition and residue of Na + timber block; (**b**) ignition and residue of composite with Palonot FR.

With regards to comparison of FR composites to Na, pHRR significantly decreases by 74% and 87% for NaP_2_ and NaP_4_, respectively. Besides, the flame height of Na composites was substantially higher Figure 6a compared to variant with FR (Figure 6b). The t_ig_ of P_2_ and P_4_ composites under piloted ignition was identical to that achieved in self-ignition. In the case of the batches with P_4_, a new peak at about 560 s is formed that corresponds to an ignition time of 493 s for one of the replicates. While this outcome can be associated with the woody nature of a biobased composite, the implication of fabrication technique is more attributable since the composite is composed of top layers with FR and core layers without FR treatment. During the test, a coat of char builds up on the exposed surface, impeding combustion and resulting in a slower rate of heat release. In some cases, such a char layer later develops fissures that allow heat radiation to reach the deeper layers of the composites, leading to ignition or increasing the rate of heat release, or a combination of both. NaP_2_ displayed an average piloted t_ig_ of 40 s, which was around 11 s faster than observed for the replica, which had the earliest t_ig_ during the pilotless fire test. Additionally, the nature of the curve for NaP_2_ suggests that the layers of material in the composite are more homogenized compared to NaP_4_. Regardless of the underlying variation in t_ig_ for variants with P_4_, the HRR shows the reproducibility of the process as the deviations are very modest. This is partly due to the localization and low intensity of the ignition, as shown in Figure 6b.

**Figure 7:**
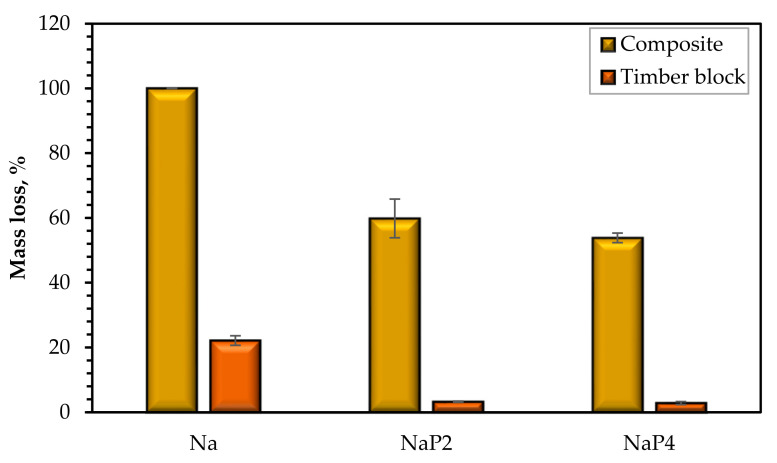
Mass loss of the composite and timber block.

Table 4 displays a summary of the piloted cone calorimeter test. Additionally, Figure 7 shows the mass loss of composites and the corresponding timber blocks. Regardless of the decline in pHRR and THR identified for NaP_2_ and NaP_4_, TSR was significantly high compared to the outcome for Na, which is attributed to incomplete combustion of the FR components in the gas phase, agreeing with the findings by Sag et al. [28]. Nonetheless, NaP_4_ shows less TSR than NaP_2_, though the margin of error in this result is very high due to a lack of ignition or protracted delay in the combustion of the NaP_4_ specimens. Despite the high TSR of NaP_2_ and NaP_4_, the CO_2_ output was noticeably lower compared to Na. Furthermore, the CO_2_ yield (150 kg kg^−1^), TSR (5 m^2^ m^−2^) and THR (104 MJm^−2^) reported for PLA by Hapuarachchi et al. [27] suggest a comparative reduction in CO_2_ emissions with fibrous reinforcement. The direct correlation of THR values is mainly dependent on the PLA matrix, since Sag et al. [28] revealed a THR value of 72.5 MJm^−2^ for PLA with comparable test settings. The mass loss shown in Figure 7 highlights the total thermal degradation of Na following the test, causing substantial flame to spread to the underlying timber block. The Palonot FR noticeably reduced the thermal deterioration of the composites, but the mass loss is comparatively different, with NaP_2_ yielding a 10 percentage points greater mass loss than NaP_4_. However, there was no consequential impact on the thermal decomposition of the wood, which was 3.3% and 2.8% for NaP_4_ and NaP_2_, respectively.

#### 3.3.3. Fire Classification

Table 5 presents the MAHRE values of the composites. These values inform the ability of the composites to support or prevent flames from spreading to nearby objects. Based on the result, the substantial implication of the Palonot FR is observed, with the FR composites meeting the most demanding hazard level (HL3) for selected requirements in the transportation sector. However, without FR treatment, the hemp fiber-reinforced PLA will not be suitable for applications where specific fire regulations are required, since the lowest permissible MAHRE value (90 kWm^−2^) for HL1 is 44% lower than that attained by Na.

In addition, a conclusion can be drawn based on the model [29] to apply the indicative test to the scale of the single burning item (SBI) test (EN 13501-1 [30]) for the application of the composites in the building construction. In this regard, the NaP_4_ composite achieves a class B prediction because the peak heat release (pHRR) from the cone calorimeter test (see Table 4) is less than 75 kWm^−2^. Even though a further estimation is necessary for NaP_2_, the obtained result appears to suggest that a class B classification would be adequate. However, the fire growth index (FIGRA), total heat release during the test (THR600), and smoke growth rate (SMOGRA) are crucial for the complete Eurocode classification.

### 3.4. Water Sorption (Hygroscopicity)

**Figure 8:**
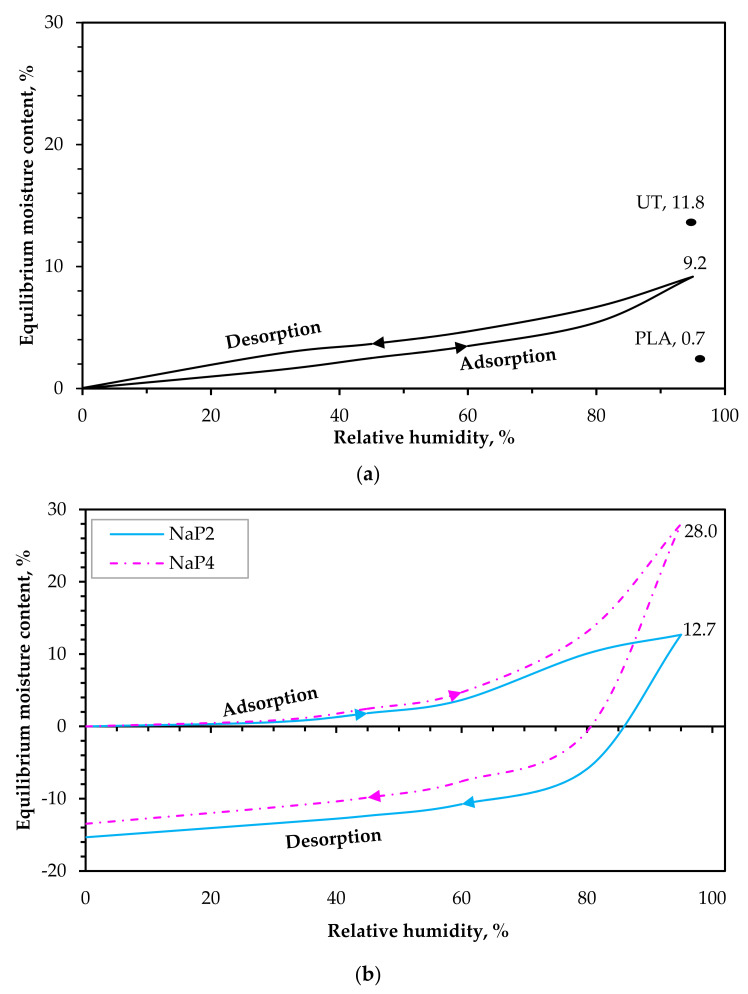
Adsorption and desorption curves of (**a**) non-FR composite (Na); (**b**) FR composite (NaP_2_ and NaP_4_).

Figure 8a shows the curve of RH and corresponding EMC (equilibrium moisture content) values for Na, while Figure 8b highlights the results for NaP_2_ and NaP_4_. For comparison, Figure 8a also includes the EMC at 95% RH for PLA and that of non-alkalized hemp fiber-reinforced PLA composite (UT) [14] for comparison. The hygroscopic behavior of the Na, especially the EMC (9.2 ± 0.01%) at RH of 95%, is similar to that published [14] for composites containing hemp fibers treated for 4 h with a solution of 5 wt.% NaOH (10.2 ± 0.4%). The comparable outcome affirms the trivial difference in the HF chemical composition when immersion for 1 h and 4 h are compared [14]. The composites display a type II sigmoidal shape of the sorption curve often reported in the literature [31] as a type of multi-molecular adsorption during which water molecules build up layer by layer, confirming the hydrophilic nature of the cellulosic composites, particularly indicating the presence of a plant fiber reinforcement. Comparing Na to the composites with Palonot FR, a substantial difference in water sorption properties is observed, with NaP_4_ resulting in significantly higher EMC (28%) at 95% RH. The desorption behavior of the FR composites is very different, with a considerable decline in desorption EMC attributed to weight loss due to the leaching (condensation of moisture and dissolution of the flame retardant) induced by an RH of 60%. This effect is because the water intake at an RH from 65% occurs due to the collection of water droplets on the surface of the composite, also reported by Pejic et al. [32]. The water activity (a_w_) during the desorption of Na samples remained roughly 1.2% higher than during adsorption. A higher EMC at desorption is usual for cellulosic materials due to the retention of cell wall moisture in the form of bound water after the fiber saturation point is attained [33]. At the end of the test, NaP_4_ indicates a 13.5% mass loss that was slightly lower than that of NaP_2_ (15.4%) but, as seen, the maximal a_w_ (28%) by NaP_4_ was substantially more than that of NaP_2_ (13%). This outcome may be due to the higher impact of mass loss for NaP_2_ before the 95% RH, since the a_w_ at 80% is not consistent with the pattern observed for Na and NaP_4_. For instance, the most substantial a_w_ during the moisture sorption occurred between an RH of 80 to 95%, with a 41.3% and 53.2% increase in water intake by Na and NaP_4_, respectively, compared to 15% by NaP_2_. However, when comparing the trend of a_w_ up to 80% for the FR composites, NaP_2_ displays a lower water uptake than NaP_4_, though a consistent trend is noted regarding the percentage increase in moisture sorption. Regardless, the FR composites produced slightly lower a_w_ values at RH values below 60% than Na. Overall, the outcome indicates that a higher moisture presence is detrimental to the integrity of the FR composite, which may impact the reaction to fire properties. Similar observations have been reported by Campana et al. [34].

## 4. Conclusions

The current work examines novel flame retardants based on protic ionic liquids on alkalized long, aligned hemp fiber-reinforced PLA composites. The flame-retardant composites were fabricated using a stacking and carded mix combination. The impact of the flame-retardant treatment on the fiber structure and composite morphology, respectively, was investigated using FTIR analysis and SEM. The response to fire and water sorption determined how well the green composite performed. One can draw the following conclusions:

1. Hemp fibers were phosphorylated by the flame-retardant treatment, and the SEM revealed flame-retardant retention and morphological changes in the FR composites because of the production process.
2. The hemp fiber/PLA green composite’s water sorption was influenced by the Palonot FR, leading to significantly greater moisture absorption, which led to the leaching of the deposited FR and concomitant weight loss.
3. With 3% mass loss as opposed to the 22% obtained for the timber covered by non-FR composite, the Palonot FR ensured considerable inhibition of flame spread to the underlying wood.
4. Among the two studied Palonot flame retardants, a batch (P_4_) is predicted to achieve a class B fire performance from the cone calorimeter test with a pHRR below 75 kWm^−2^ (50 kWm^−2^). Although the significant smoke emissions are a drawback, both FR variants demonstrate effective results for application in the transportation sector, with MAHRE values of 15–19 kWm^–2^.

The study shows that alkalized hemp fiber-reinforced PLA composites can achieve an enhanced reaction to fire performance. The suggestive test and moisture sorption highlight the limitations that need more attention before larger-scale fire experiments are carried out to validate the results.

## Figures and Tables

**Table 1:** Fabricated composite batches.

Composite	Non-FR	With FR	
		Palonot 2	Palonot 4
NaOH treated hemp fiber PLA	Na	NaP_2_	NaP_4_
Untreated hemp fiber PLA	UT	UTP_2_	UTP_4_

**Table 2:** The average mass and thickness of the composite specimens and timber blocks.

	Mass, g		Thickness, mm	
Specimen	Composites	Timber Blocks	Composites	Timber Block
Na	29.5 ± 0.4	163.6 ± 8.2	2.9 ± 0.0	25.5 ± 0.8
NaP_2_	35.6 ± 1.5	160.0 ± 0.1	2.8 ± 0.2	25.4 ± 0.1
NaP_4_	35.9 ± 2.0	160.7 ± 1.5	2.9 ± 0.1	25.3 ± 0.2

**Table 3:** Test specification for transportation applications based on the EN 45545-2.

Category	Application	Examples	Hazard Level
Requirement (R)1	INIA: Interior vertical surfaces	Walls Partitions Hoods	HL1 * HL2 = 90 kWm^−2^ HL3 = 60 kWm^−2^
	INIB: Interior horizontal downward facing surfaces	Ceiling panels Boxes	
	INID: Horizontal/vertical surfaces INIC: Horizontal/upward facing components. IN7: Window surroundings	Window frames	
R2	IN2, IN9A, and IN10:	Tables, containers	HL1 * and HL2 * HL3 = 90 kWm^−2^
R6	F1C, FID	Seat and back shells	HL1 = 90 kWm^−2^, HL2 = 90 kWm^−2^, HL3 = 60 kWm^−2^
R19 and R21	Heat flux (25 kWm^−2^)	Seats	HL1 = 75 kWm^−2^, HL2 = 50 kWm^−2^, HL3 = 50 kWm^−2^

* Does not qualify.

**Table 4:** Parameters from the piloted ignition cone calorimeter test.

Sample	Peak HRR, kWm^−2^	THR, MJm^−2^	TSR, m^2^ m^−2^	Mean CO_2_, kgkg^−1^
Na	292 ± 7.1	83 ± 5.2	75 ± 5.5	1.5 ± 0.0
NaP_2_	77 ± 7.4	7.1 ± 0.5	189 ± 25.6	0.7 ± 0.1
NaP_4_	50 ± 5.2	6.1 ± 1.5	123 ± 163	0.9 ± 0.3

**Table 5:** Classification of reaction to fire based on EN 45545-2.

Composites	MAHRE, kWm^−2^
Na	162 ± 5.7
NaP_2_	19 ± 3.4
NaP_4_	15 ± 2.2

## Data Availability

Data are available on request due to some restrictions of the project privacy.

## Supplementary Materials

The following supporting information can be downloaded at: https://www.mdpi.com/article/10.3390/polym15183661/s1, Figure S1: Scanning electron microscope image identifying spectra 1–4 (a); (b) Energy dispersive X-ray (EDS) spectrograph of untreated hemp fiber-reinforced polylactic acid (PLA) composite (UT); Figure S2: SEM identifying EPOXY section observed on the composites; Figure S3: (a) SEM image showing atomic composition (spectra 1–7) of the alkalized hemp fiber-reinforced PLA composite (Na); (b) EDS spectrograph of Na; Figure S4: SEM image (a) identifying the elemental composition (spectra 1–4); corresponding EDS spectrograph (b) of untreated hemp fiber-reinforced composites containing Palonot 4 (UTP_4_); Figure S5: SEM image identifying the elemental composition (spectra 1–9) (a); corresponding EDS spectrograph (b) of alkalized hemp fiber-reinforced PLA composite containing Palonot 4 (NaP_4_); Table S1: EDS analysis corresponding to Figure S1 (spectra 1–4); Table S2: EDS analysis corresponding to Figure S2; Table S3: EDS analysis corresponding to Figure S3 (spectra 1–7); Table S4: EDS analysis corresponding to Figure S4 (spectra 1–7); Table S5: EDS analysis corresponding to Figure S5 (spectra 1–9).
