# Supplementary material for: Influence of Protic Ionic Liquid-Based Flame Retardant on the Flammability and Water Sorption of Alkalized Hemp Fiber-Reinforced PLA Composites
Source: Polymers (Basel). 2023 Sep 5;15(18):3661. doi: 10.3390/polym15183661 (PMC10534385; doi:10.3390/polym15183661)
Supplement: Supplementary file 1 [file polymers-15-03661-s001.zip › polymers-2589934-supplementary.pdf]

Energy-dispersive X-ray spectroscopy report

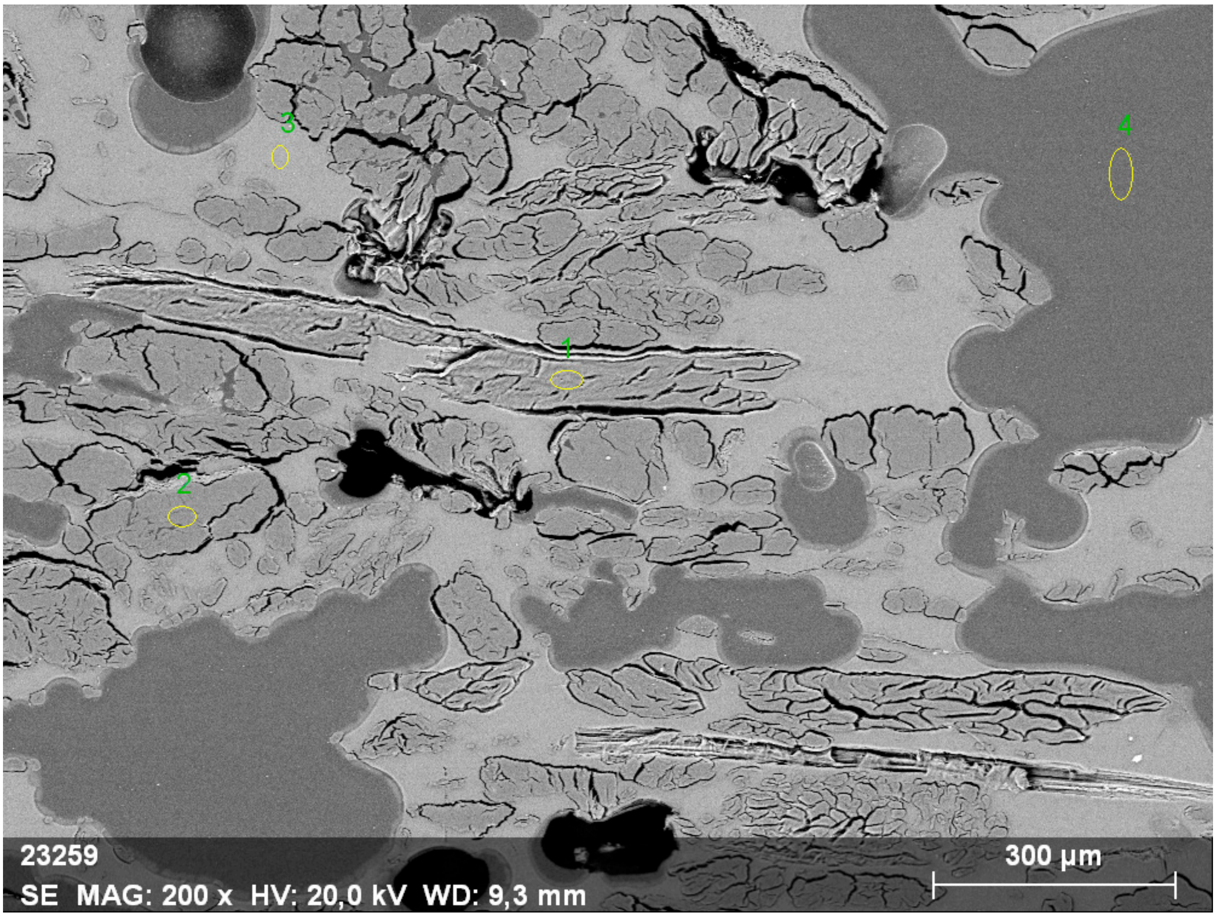

(a)

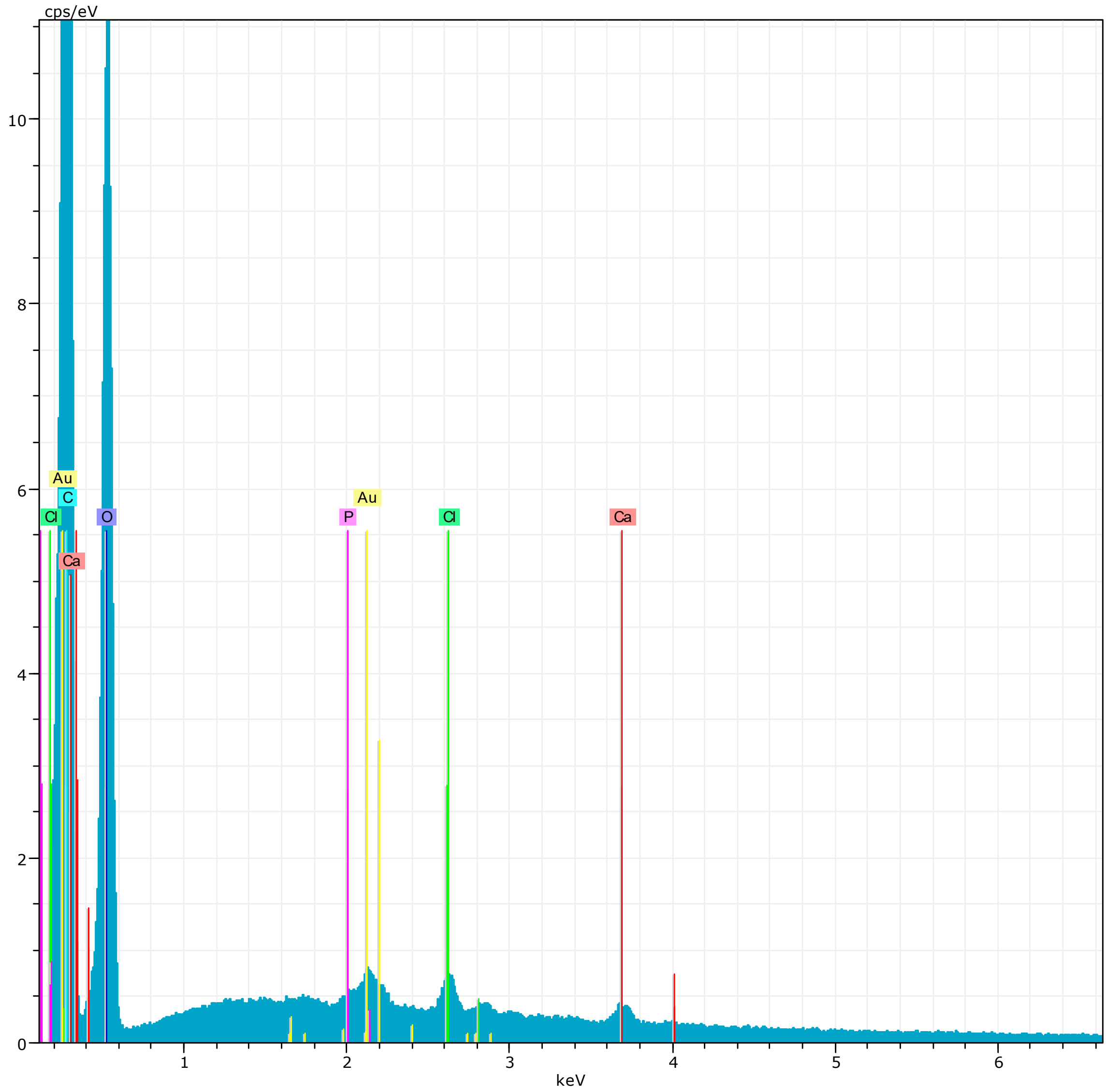

(b)

Figure S1: Scanning electron microscope image identifying spectrums 1-4 (a); (b) Energy dispersive X-ray (EDS) spectrograph of untreated hemp fiber reinforced polylactic acid (PLA) composite (UT).

Table S1: EDS analysis corresponding to Figure S1 (Spectrum 1-4).

Spectrum: 1

Element	Series	unn. C [wt.%]	norm. C [wt.%]	Atom. C [at.%]	Error [%]
Calcium	K-series	0.31	0.31	0.11	0.0
Oxygen	K-series	55.27	55.27	48.25	17.3
Carbon	K-series	44.41	44.41	51.64	13.8
	Total:	100.00	100.00	100.00	

Spectrum: 2

Element	Series	unn. C	norm. C	Atom. C	Error

		[wt.%]	[wt.%]	[at.%]	[%]
Calcium	K-series	0.35	0.35	0.12	0.0
Oxygen	K-series	53.78	53.78	46.75	16.9
Carbon	K-series	45.87	45.87	53.12	14.3
	Total:	100.00	100.00	100.00	

Spectrum: 3

Element	Series	unn. C [wt.%]	norm. C [wt.%]	Atom. C [at.%]	Error [%]
Oxygen	K-series	36.73	36.73	30.35	11.7
Carbon	K-series	63.27	63.27	69.65	19.4
	Total:	100.00	100.00	100.00	

Spectrum: 4 EPOXY

Element	Series	unn. C [wt.%]	norm. C [wt.%]	Atom. C [at.%]	Error [%]
Chlorine	K-series	1.57	1.57	0.60	0.1
Phosphorus	K-series	0.28	0.28	0.12	0.0
Oxygen	K-series	36.06	36.06	30.53	11.9
Carbon	K-series	60.88	60.88	68.66	20.4
	Total:	100.00	100.00	100.00	

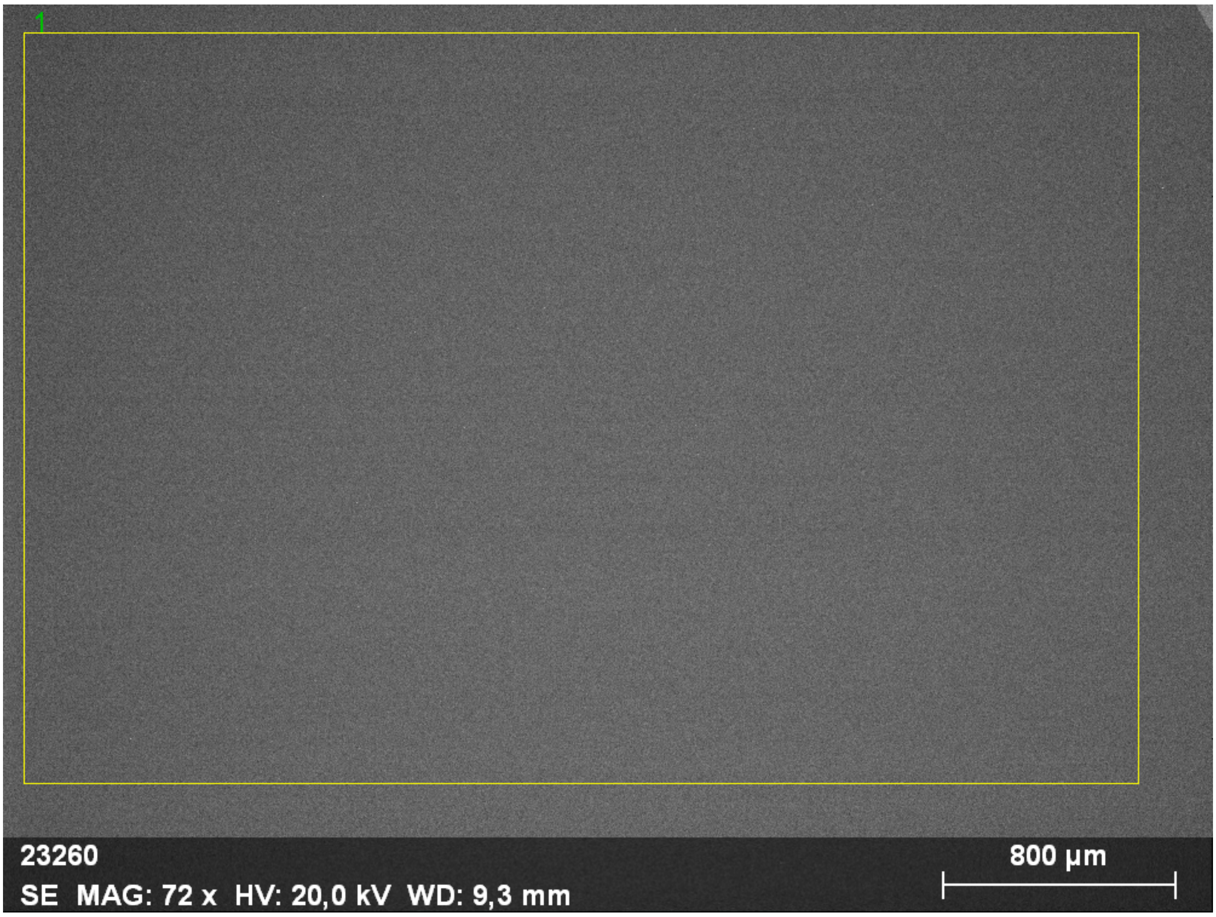

Figure S2: SEM identifying EPOXY section observed on the composites.

Table S2: EDS analysis corresponding to Figure S2.

Spectrum: 1

Element	Series	unn. C [wt.%]	norm. C [wt.%]	Atom. C [at.%]	Error [%]
Chlorine	K-series	1.46	1.46	0.56	0.1
Phosphorus	K-series	0.31	0.31	0.14	0.0
Oxygen	K-series	38.46	38.46	32.74	12.6
Carbon	K-series	58.62	58.62	66.48	19.6
	Total:	100.00	100.00	100.00	

Note: Prior to coating and SEM imaging, the composite samples are glued to the stud using epoxy resin.

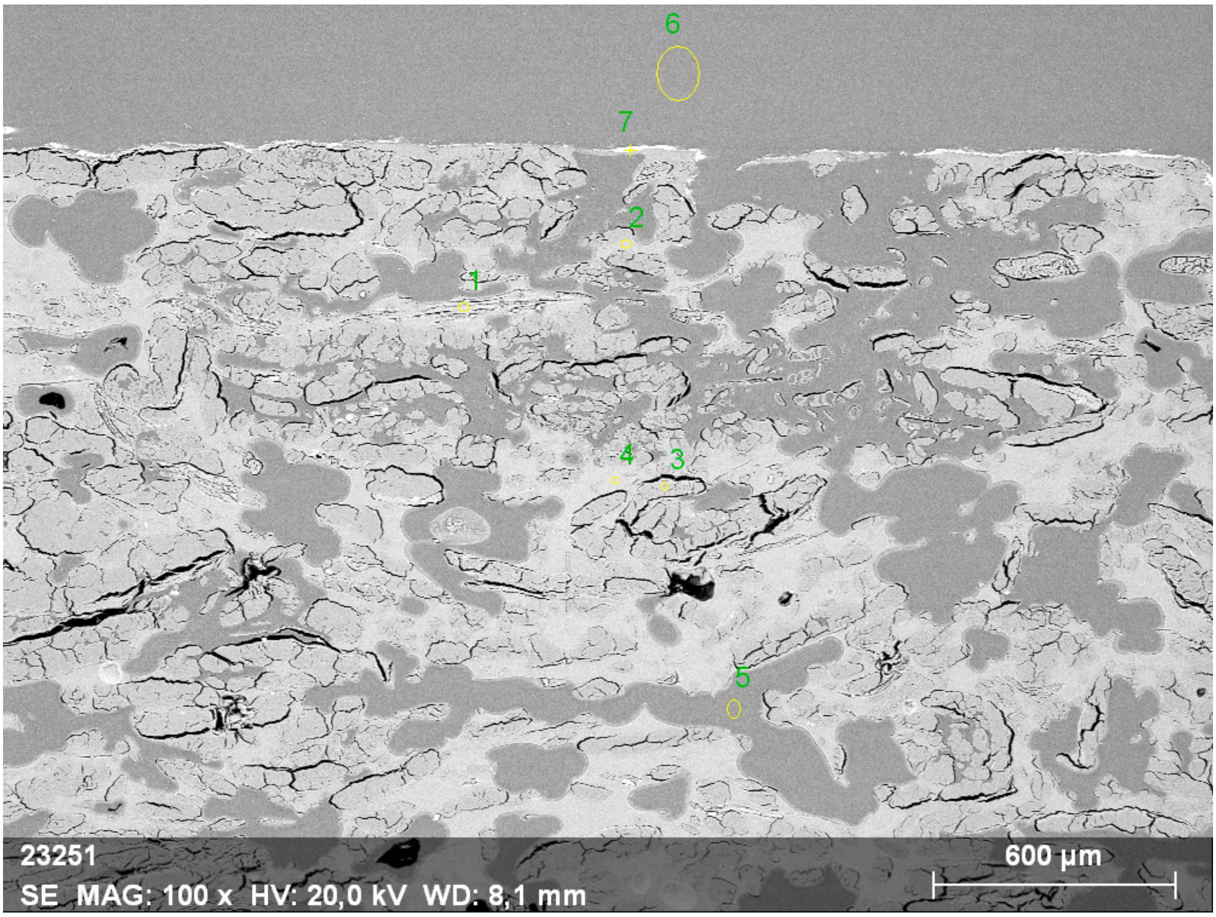

(a)

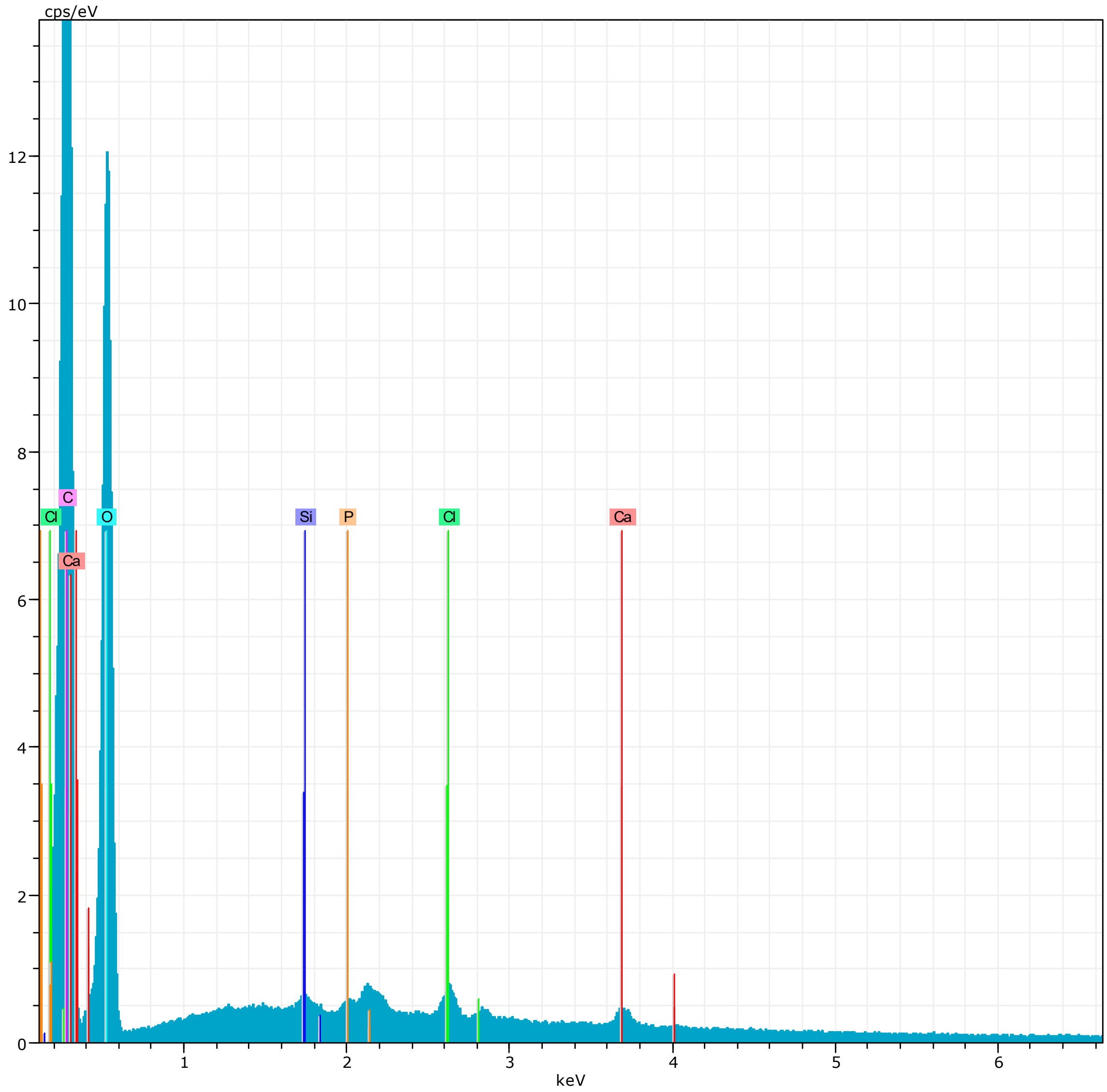

(b)

Figure S3: (a) SEM image showing atomic composition (spectrums 1-7) of the alkalized hemp fiber reinforced PLA composite (Na); (b) EDS spectrograph of Na.

Table S3: : EDS analysis corresponding to Figure S3 (Spectrum 1- 7).

Spectrum 1

Element	Series	unn. C [wt.%]	norm. C [wt.%]	Atom. C [at.%]	Error [%]
Calcium	K-series	0.27	0.27	0.09	0.0
Silicon	K-series	0.18	0.18	0.09	0.0
Oxygen	K-series	43.23	43.23	36.49	13.7
Carbon	K-series	56.32	56.32	63.33	17.4
	Total:	100.00	100.00	100.00	

Spectrum: 2

Element	Series	unn. C [wt.%]	norm. C [wt.%]	Atom. C [at.%]	Error [%]

Silicon	K-series	0.11	0.11	0.05	0.0
Oxygen	K-series	41.15	41.16	34.45	13.0
Carbon	K-series	58.74	58.74	65.50	18.0
	Total:	100.00	100.00	100.00	

Spectrum: 3

Element	Series	unn. C [wt.%]	norm. C [wt.%]	Atom. C [at.%]	Error [%]
Calcium	K-series	0.58	0.58	0.20	0.0
Oxygen	K-series	51.04	51.04	44.13	16.1
Carbon	K-series	48.30	48.30	55.63	15.0
Silicon	K-series	0.09	0.09	0.04	0.0
	Total:	100.00	100.00	100.00	

Spectrum: 4

Element	Series	unn. C [wt.%]	norm. C [wt.%]	Atom. C [at.%]	Error [%]
Oxygen	K-series	37.03	37.03	30.63	11.8
Carbon	K-series	62.97	62.97	69.37	19.3
	Total:	100.00	100.00	100.00	

Spectrum: 5 **EPOXY**

Element	Series	unn. C [wt.%] [	norm. C wt.%]	Atom. C [at.%]	Error [%]
Chlorine	K-series	1.52	1.52	0.58	0.1
Phosphorus	K-series	0.26	0.26	0.11	0.0
Oxygen	K-series	33.74	33.74	28.34	11.2
Carbon	K-series	63.35	63.35	70.89	21.2
	Total:	100.00	100.00	100.00	

Spectrum: 6 EPOXY

Element	Series	unn. C [wt.%]	norm. C [wt.%]	Atom. C [at.%]	Error [%]
Chlorine	K-series	1.45	1.45	0.55	0.10
Phosphorus	K-series	0.26	0.26	0.12	0.00
Oxygen	K-series	37.30	37.31	31.60	12.2
Carbon	K-series	59.96	59.97	67.66	20.1
	Total:	100.00	100.00	100.00	

Spectrum: 7 metal coating on the surface before casting in EPOXY

Element	Series	unn. C [wt.%]	norm. C [wt.%]	Atom. C [at.%]	Error [%]
Silver	L-series	40.65	38.82	7.40	1.3
Oxygen	K-series	29.83	28.49	36.62	5.0
Carbon	K-series	34.23	32.69	55.98	4.5

Total:	104.70	100.00	100.00

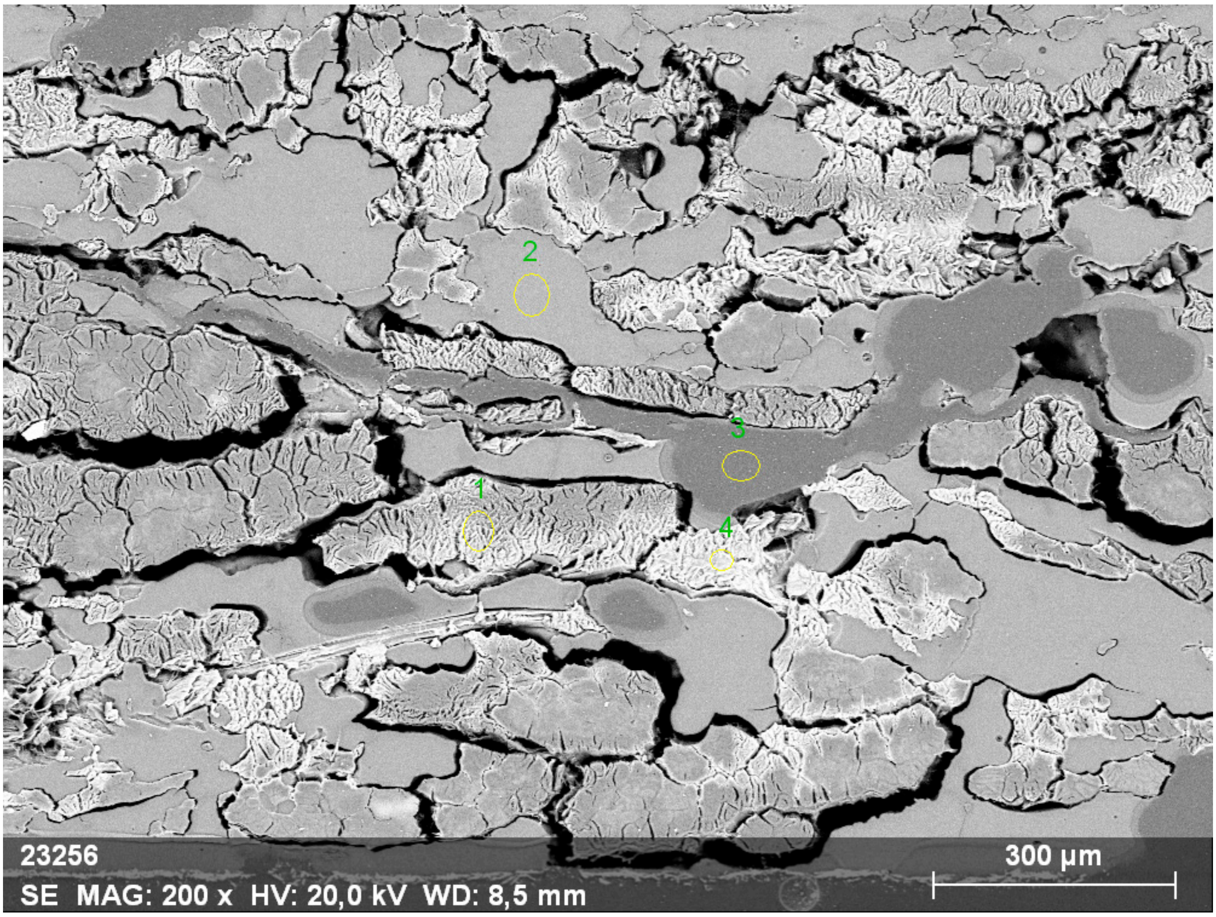

(a)

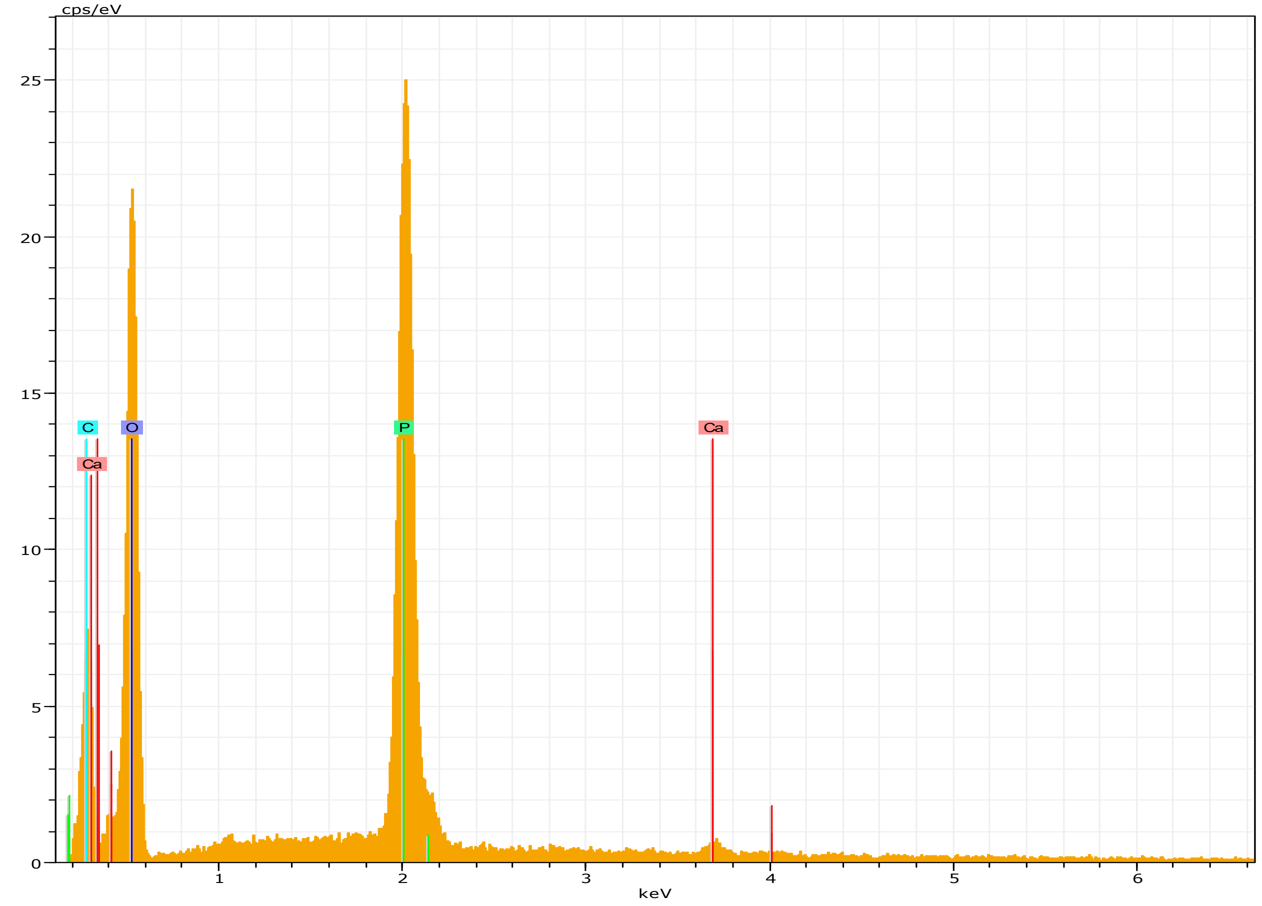

(b)

Figure S4: SEM image (a) identifying the elemental composition (spectrum 1-4); corresponding EDS spectrograph (b) of untreated hemp fiber reinforced composites containing Palonot 4 (UTP_4).

Table S4: EDS analysis corresponding to Figure S4 (Spectrum 1-7).

Spectrum: 1

Element	Series	unn. C [wt.%]	norm. C [wt.%]	Atom. C [at.%]	Error [%]
Calcium	K-series	0.38	0.38	0.14	0.0
Phosphorus	K-series	6.02	6.02	2.93	0.3
Oxygen	K-series	66.10	66.11	62.37	20.9
Carbon	K-series	27.50	27.50	34.56	9.1
	Total:	100.00	100.00	100.00	

Spectrum: 2

Element	Series	unn. C [wt.%]	norm. C [wt.%]	Atom. C [at.%]	Error [%]
Oxygen	K-series	46.60	46.60	39.58	14.6
Carbon	K-series	53.40	53.40	60.42	16.4
	Total:	100.00	100.00	100.00	

Spectrum: 3

Element	Series	unn. C [wt.%]	norm. C [wt.%]	Atom. C [at.%]	Error [%]
Chlorine	K-series	1.49	1.49	0.57	0.1
Phosphorus	K-series	0.28	0.28	0.12	0.0
Oxygen	K-series	37.16	37.16	31.50	12.2
Carbon	K-series	60.00	60.00	67.73	20.1
	Total:	100.00	100.00	100.00	

Spectrum: 4

Element	Series	unn. C [wt.%]	norm. C [wt.%]	Atom. C [at.%]	Error [%]
Calcium	K-series	0.55	0.55	0.22	0.1
Phosphorus	K-series	12.57	12.57	6.54	0.5
Oxygen	K-series	69.59	69.59	70.06	22.3
Carbon	K-series	17.28	17.28	23.18	6.3
	Total:	100.00	100.00	100.00	

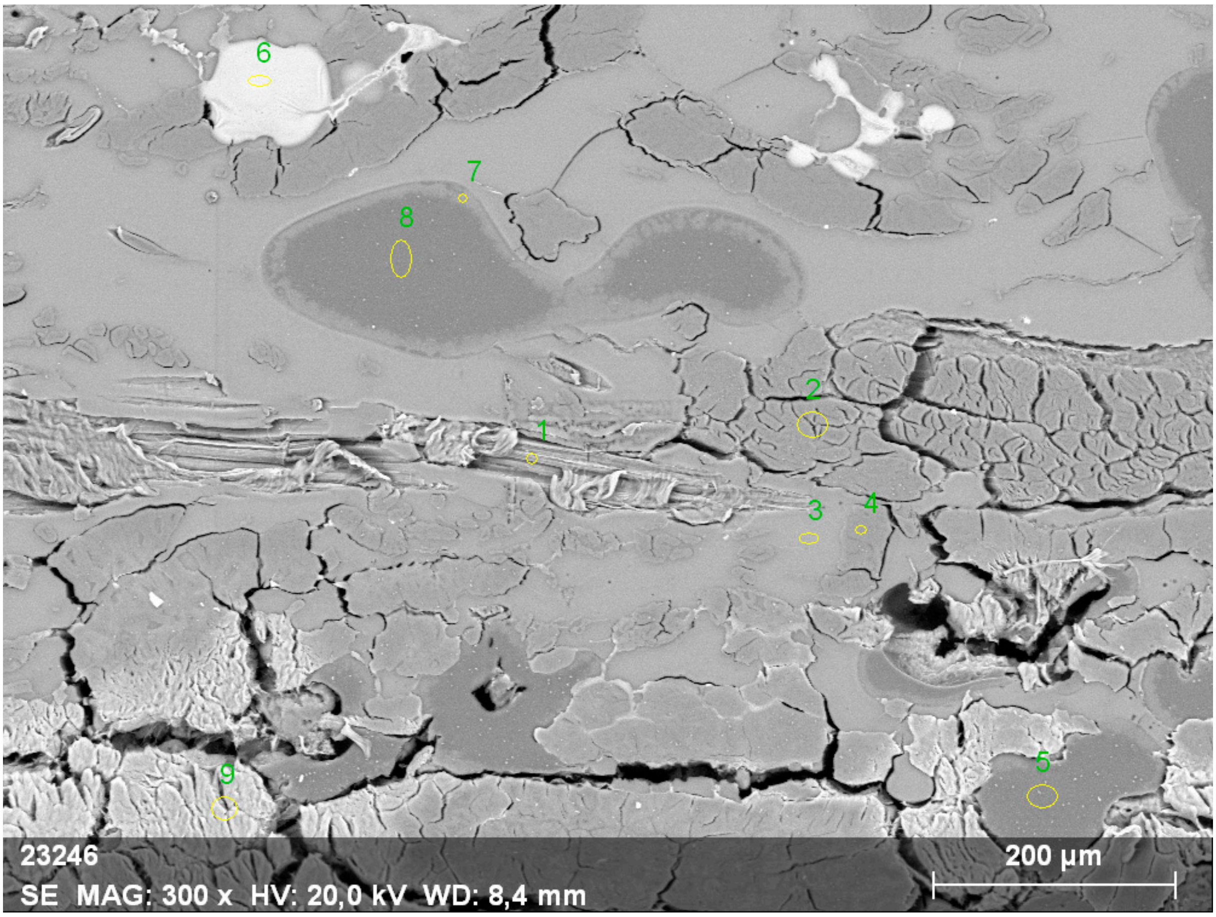

(a)

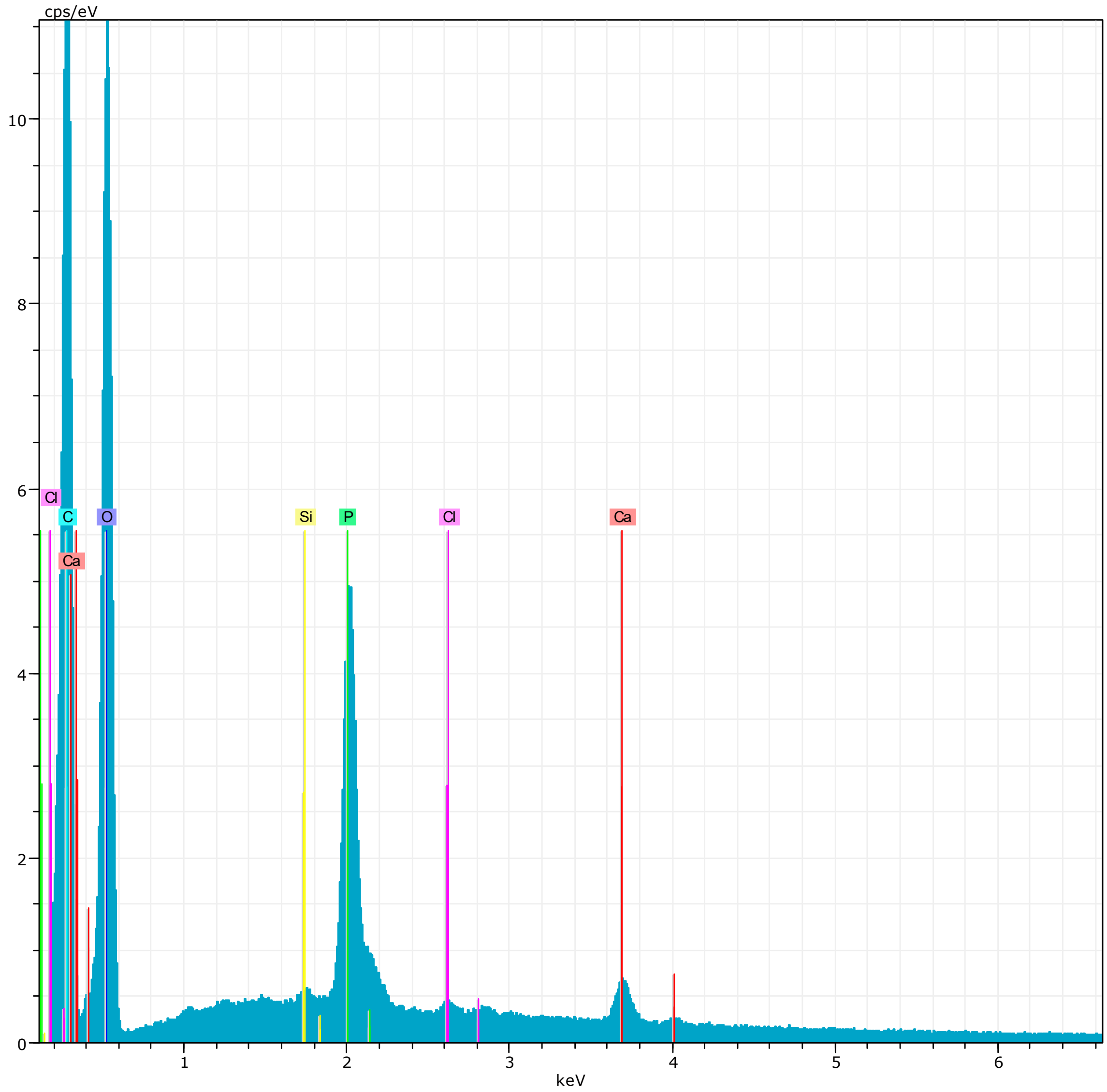

(b)

Figure S5: SEM image identifying the elemental composition (spectrums 1-9) (a); corresponding EDS spectrograph (b) of alkalized hemp fiber reinforced PLA composite containing Palonot 4 (NaP_4).

Table S5: EDS analysis corresponding to Figure S5 (Spectrum 1 – 9).

Spectrum: 1

Element	Series	unn. C [wt.%]	norm. C [wt.%]	Atom. C [at.%]	Error [%]
Calcium	K-series	0.70	0.70	0.25	0.1
Phosphorus	K-series	0.89	0.89	0.41	0.1
Oxygen	K-series	57.38	57.38	51.69	18.4
Carbon	K-series	39.61	39.61	47.54	13.4
	Total:	100.00	100.00	100.00	

Spectrum: 2

Element	Series	unn. C [wt.%]	norm. C [wt.%]	Atom. C [at.%]	Error [%]

Calcium	K-series	0.88	0.88	0.32	0.1
Phosphorus	K-series	0.37	0.37	0.17	0.1
Oxygen	K-series	60.92	60.92	55.40	19.4
Carbon	K-series	36.31	36.31	43.99	12.3
	Total:	100.00	100.00	100.00	

Spectrum: 3

Element	Series	unn. C [wt.%]	norm. C [wt.%]	Atom. C [at.%]	Error [%]
Oxygen	K-series	33.26	33.26	27.23	10.6
Carbon	K-series	66.74	66.74	72.77	20.5
	Total:	100.00	100.00	100.00	

Spectrum: 4

Element	Series	unn. C [wt.%]	norm. C [wt.%]	Atom. C [at.%]	Error [%]
Calcium	K-series	0.31	0.31	0.11	0.0
Carbon	K-series	52.64	52.64	59.78	16.3
Oxygen	K-series	47.05	47.05	40.11	14.8
	Total:	100.00	100.00	100.00	

Spectrum: 5 EPOXY

Element	Series	unn. C [wt.%]	norm. C [wt.%]	Atom. C [at.%]	Error [%]
Chlorine	K-series	1.53	1.53	0.58	0.1
Oxygen	K-series	35.40	35.40	29.92	11.7
Carbon	K-series	61.54	61.54	69.29	20.6
Phosphorus	K-series	0.30	0.30	0.13	0.0
	Total:	100.00	100.00	100.00	

Spectrum: 6

Element	Series	unn. C [wt.%]	norm. C [wt.%]	Atom. C [at.%]	Error [%]
Calcium	K-series	1.21	1.21	0.51	0.1
Phosphorus	K-series	15.70	15.70	8.60	0.6
Oxygen	K-series	75.23	75.23	79.78	24.4
Carbon	K-series	7.87	7.87	11.12	3.7
	Total:	100.00	100.00	100.00	

Spectrum: 7

Element	Series	unn. C [wt.%]	norm. C [wt.%]	Atom. C [at.%]	Error [%]
Chlorine	K-series	1.06	1.06	0.41	0.10
Oxygen	K-series	37.32	37.32	31.75	12.2

Carbon	K-series	59.68	59.68	67.64	19.8
Phosphorus	K-series	0.17	0.17	0.07	0.0
	Total:	100.00	100.00	100.00	

Spectrum: 8 EPOXY

Element	Series	unn. C [wt.%]	norm. C [wt.%]	Atom. C [at.%]	Error [%]
Chlorine	K-series	1.52	1.52	0.58	0.1
Oxygen	K-series	35.69	35.69	30.18	11.8
Carbon	K-series	61.28	61.28	69.03	20.6
Phosphorus	K-series	0.29	0.29	0.13	0.0
	Total:	100.00	100.00	100.00	

Spectrum: 9

Element	Series	unn. C [wt.%]	norm. C [wt.%]	Atom. C [at.%]	Error [%]
Phosphorus	K-series	11.66	11.66	6.00	0.5
Calcium	K-series	1.25	1.25	0.50	0.1
Oxygen	K-series	66.84	66.84	66.61	21.5
Carbon	K-series	20.26	20.26	26.89	7.2
	Total:	100.00	100.00	100.00	
